# Evaluating the Clinical Outcomes of Empagliflozin in Heart Failure Patients With Preserved Ejection Fraction: A Systematic Review

**DOI:** 10.7759/cureus.84026

**Published:** 2025-05-13

**Authors:** Saba Sattar, Mansi Yadav, Ankitakumari B Maisuriya, Olamide Ogunfunwa, Ahmad Bin Abdul Qayyum Satti, Husnain Ali, Muhammad Muaz Mushtaq, Saba Amjad, Azlaan Hussain, Nikhil Deep Kolanu

**Affiliations:** 1 Medicine, King Edward Medical University, Lahore, PAK; 2 Internal Medicine, Pandit Bhagwat Dayal Sharma Post Graduate Institute of Medical Sciences, Rohtak, IND; 3 Medicine and Surgery, Government Medical College, Surat, Surat, IND; 4 Internal Medicine, Federal Medical Center Abeokuta, Abeokuta, NGA; 5 Internal Medicine, King Edward Medical University, Lahore, PAK; 6 Medicine and Surgery, King Edward Medical University, Lahore, PAK; 7 Obstetrics and Gynaecology, The Aga Khan Hospital, Karachi, PAK; 8 Medicine and Surgery, Mayo Hospital, Lahore, PAK; 9 Internal Medicine, China Medical University, Shenyang, CHN

**Keywords:** diabetes, empaglifozin, emperial, emperor, heart failure, hfpef, sglt 2 inhibitor

## Abstract

Heart failure with preserved ejection fraction (HFpEF) represents a significant clinical challenge due to its complex pathophysiology and limited therapeutic options. This systematic review evaluates the efficacy and safety of empagliflozin, a sodium-glucose co-transporter 2 (SGLT2) inhibitor, in patients with HFpEF. Following Preferred Reporting Items for Systematic Reviews and Meta-Analyses (PRISMA) guidelines, we analyzed 11 studies, predominantly from the EMPEROR-Preserved trial and its sub-analyses, investigating empagliflozin in HFpEF patients. Our findings consistently demonstrate that empagliflozin significantly reduces heart failure hospitalizations (HR 0.71, 95% CI 0.60-0.83) across diverse patient populations. This benefit was observed regardless of sex, age, diabetes status, blood pressure, and frailty levels, although effects were attenuated in patients with higher ejection fractions and in severely frail individuals. Notably, empagliflozin did not significantly reduce cardiovascular mortality, although a non-significant trend toward benefit was observed in certain subgroups. Additional benefits included slowed decline in renal function and improved quality of life measures. The safety profile was favorable across all studies, with good tolerability even in elderly and frail populations. These findings position empagliflozin as a valuable addition to the limited therapeutic armamentarium for HFpEF, particularly for reducing hospitalizations and improving symptoms, though its impact on mortality appears limited. Future research should focus on identifying specific HFpEF phenotypes that derive maximal benefit from empagliflozin and evaluating its long-term efficacy, optimal combination strategies, and real-world effectiveness across diverse populations.

## Introduction and background

Heart failure with preserved ejection fraction (HFpEF) is a complex and heterogeneous clinical syndrome characterized by symptoms of heart failure, left ventricular ejection fraction (LVEF) ≥50%, and evidence of diastolic dysfunction or elevated filling pressures [1]. The estimated incidence of HFpEF is approximately 27 cases per 10,000 person-years, with a significant increase observed over the past two decades [1]. Unlike heart failure with reduced ejection fraction (HFrEF), for which multiple evidence-based therapies have been established, treatment options for HFpEF remain limited, with most interventions focusing on symptom relief rather than modifying disease progression, which in HFpEF typically includes worsening New York Heart Association (NYHA) functional class, increased hospitalization frequency, and reduced exercise tolerance. The lifetime risk of HFpEF at age 45 exceeds 10% in both men and women, highlighting its growing public health burden [1]. Notably, HFpEF is more prevalent among women, with hospitalizations occurring at nearly twice the rate seen in men. Racial disparities also exist, with Black women experiencing the highest hospitalization rates for HFpEF compared to other race-sex groups. These epidemiological trends underscore the need for heightened clinical awareness and improved management strategies for HFpEF [1-3].

Empagliflozin, a sodium-glucose co-transporter 2 (SGLT2) inhibitor, was originally developed for glycemic control in patients with type 2 diabetes mellitus (T2DM). However, emerging evidence has demonstrated its cardiovascular benefits beyond glycemic control, particularly in the management of heart failure [4]. The EMPA-REG OUTCOME trial was the first large-scale study to reveal the cardioprotective effects of empagliflozin, showing significant reductions in cardiovascular mortality and hospitalization for heart failure among diabetic patients [5]. These findings paved the way for further investigation into the role of SGLT2 inhibitors in heart failure populations, regardless of diabetic status.

The EMPEROR-Preserved trial, a landmark randomized controlled trial, specifically evaluated the efficacy of empagliflozin in HFpEF patients [6]. This trial demonstrated a significant reduction in the composite endpoint of cardiovascular death or heart failure hospitalization with empagliflozin compared to placebo, leading to its approval as a novel therapy for HFpEF. The underlying mechanisms by which empagliflozin exerts its beneficial effects in HFpEF remain an area of active research, with proposed mechanisms including natriuresis, osmotic diuresis, reduction in preload and afterload, anti-inflammatory effects, mitochondrial efficiency, and improved cardiac energetics. Despite these promising findings, real-world data and further systematic analyses are required to consolidate the benefits of empagliflozin in diverse HFpEF populations, including those with varying degrees of left ventricular dysfunction, renal impairment, and comorbid conditions. Additionally, understanding its comparative efficacy with other emerging HFpEF therapies, such as mineralocorticoid receptor antagonists, angiotensin receptor-neprilysin inhibitors, and glucagon-like peptide-1 (GLP-1) receptor agonists, is crucial for optimizing treatment strategies.

This systematic review aims to comprehensively evaluate the clinical outcomes associated with empagliflozin in patients with HFpEF. By synthesizing data from randomized controlled trials, observational studies, and meta-analyses, we aim to provide a detailed understanding of its impact on cardiovascular mortality, heart failure hospitalizations, symptom burden, quality of life, and safety profile. Through this analysis, we seek to clarify the role of empagliflozin in contemporary HFpEF management and identify potential areas for future research.

## Review

Materials and methods

This systematic review is reported in accordance with the Preferred Reporting Items for Systematic Reviews and Meta-Analyses (PRISMA) 2020 guidelines to ensure a rigorous and comprehensive evaluation of the included studies on the outcomes of empagliflozin in HFpEF [7].

Search Strategy

A comprehensive literature search was conducted across four databases: MEDLINE (via PubMed), Embase, Cochrane Central Register of Controlled Trials (CENTRAL), and Web of Science, from January 1, 2020, to January 1, 2025. The search strategy incorporated keywords and Medical Subject Headings (MeSH) related to the population, such as "heart failure with preserved ejection fraction" and "HFpEF"; the intervention, including "empagliflozin" and "SGLT2 inhibitor"; and the outcomes, such as "cardiovascular mortality," "heart failure hospitalization," and "quality of life." To ensure a comprehensive search, reference lists of relevant reviews and included studies were also manually screened to identify any additional publications that may not have been captured in the initial database search.

Eligibility Criteria

Studies were included if they involved adults aged 18 years or older diagnosed with HFpEF, with or without diabetes. The intervention of interest was empagliflozin, either as monotherapy or in combination with standard heart failure therapy. Comparators included placebo, no treatment, or other heart failure medications, with the exception of other SGLT2 inhibitors. The primary outcomes assessed were cardiovascular mortality, heart failure hospitalizations, and changes in left ventricular function. Secondary outcomes included changes in exercise capacity, symptom burden, and safety outcomes, such as adverse events. Eligible study designs consisted of randomized controlled trials (RCTs) and observational studies, including cohort and case-control studies. Case reports, case series, studies focusing on patients with HFrEF, and those solely evaluating glycemic control or non-cardiac outcomes were excluded. Additionally, animal studies, in vitro experiments, reviews, editorials, conference abstracts, and non-English publications were not considered.

Study Selection

Two independent reviewers (M.Y. and O.O.) conducted the study selection process. Initially, titles and abstracts of all retrieved records were screened to exclude clearly irrelevant studies. Full-text articles were then obtained for all potentially eligible studies and reviewed independently by both reviewers. Any discrepancies in study inclusion were resolved through discussion, and if necessary, a third reviewer (S.S.) was consulted to reach consensus.

Data Extraction

Data were extracted independently by two reviewers (H.A. and A.H.) using a predesigned, piloted Microsoft Excel (Redmond, WA, USA) form. Extracted information included study characteristics such as author name, year of publication, country, study design, setting, and duration. Participant demographics, including age, sex, and baseline characteristics, were also recorded. Key findings and conclusions from each study were systematically documented to ensure comprehensive data collection.

Data Synthesis

Given the heterogeneity in study designs, patient populations, intervention protocols, and outcome measures, a narrative synthesis was performed. Findings were summarized in descriptive tables that outlined key study characteristics and results. A qualitative analysis was conducted to integrate and contextualize the outcomes within current clinical practice and future research directions. Due to variability among the included studies, a meta-analysis was not conducted, as pooling of results was not feasible.

This rigorous methodology ensures a transparent and comprehensive evaluation of the clinical outcomes of empagliflozin in HFpEF, providing a strong evidence base for clinical decision-making.

Results

Study Selection Process

**Figure 1:**
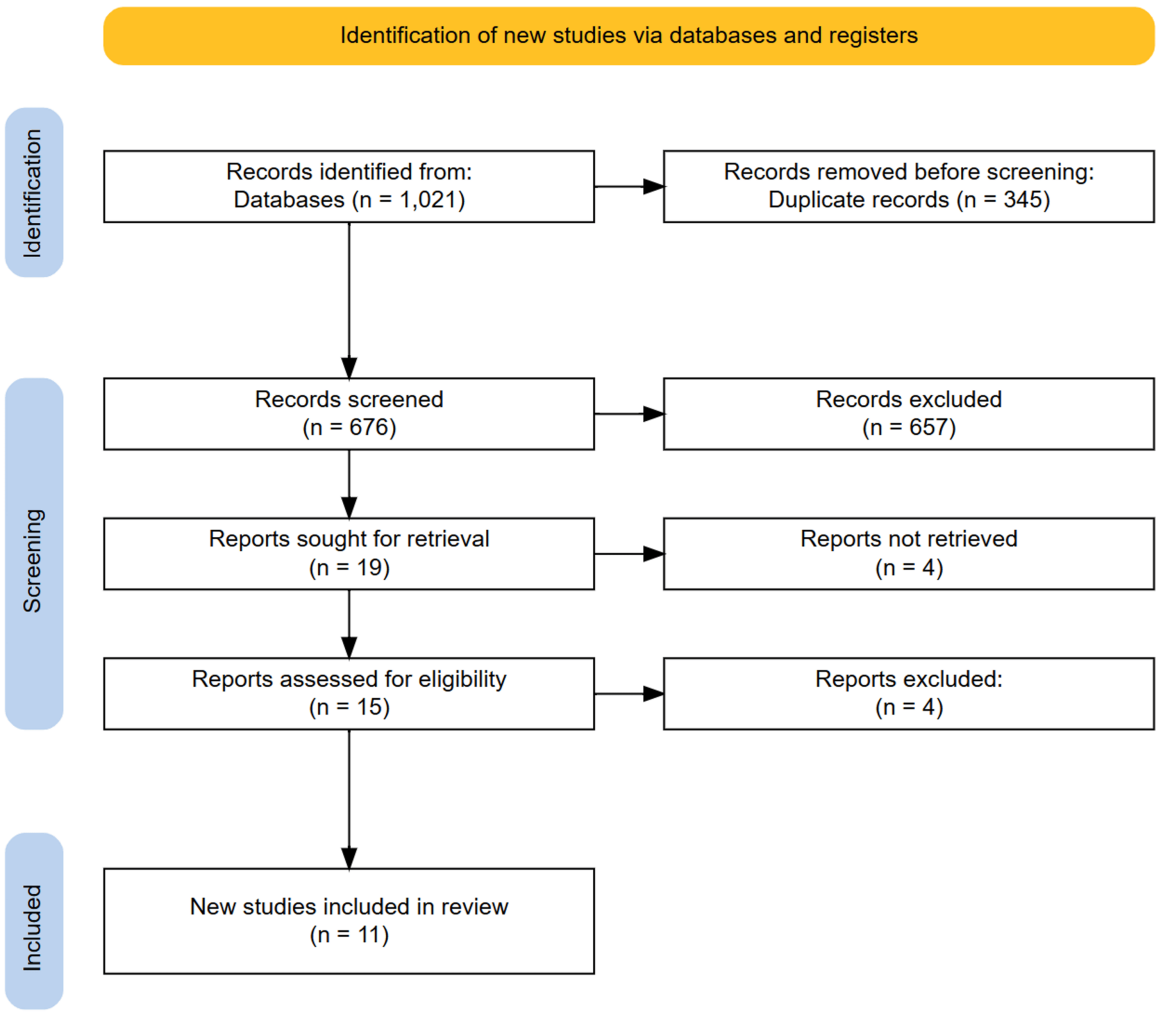
Preferred Reporting Items for Systematic Reviews and Meta-Analyses (PRISMA) diagram showing the study selection process.

Following a comprehensive database search, 1,021 articles were initially identified. After removing 345 duplicates, the titles and abstracts of the remaining 676 studies were screened, leading to the exclusion of 657 articles that did not meet the inclusion criteria. Subsequently, 19 full-text articles were assessed for eligibility, out of which 11 met the predefined inclusion criteria. The reference lists of these selected articles were further examined, but no additional relevant studies were identified. The study selection process, conducted in adherence to PRISMA guidelines, is illustrated in the PRISMA flowchart (Figure 1).

Study Characteristics

The systematic review included 11 studies investigating the effects of empagliflozin in patients with HFpEF [6,8-17]. All studies defined preserved ejection fraction as LVEF > 40% (Table 1). The majority of the included studies were RCTs, with most being part of or derived from the EMPEROR-Preserved trial (n=10), which enrolled 5,988 patients randomized to empagliflozin (n=2,997) or placebo (n=2,991). Follow-up periods ranged from 12 weeks in shorter trials to a median of 26.2 months in the larger EMPEROR-Preserved studies. The EMPERIAL-Preserved trial specifically examined exercise capacity with a smaller sample of 315 HFpEF patients [8].

**Table 1:** Study characteristics of included studies. RCT: Randomized Controlled Trial, LVEF: Left Ventricular Ejection Fraction, CV: Cardiovascular, HF: Heart Failure, EF: Ejection Fraction, HR: Hazard Ratio, CI: Confidence Interval, eGFR: estimated Glomerular Filtration Rate, MRA: Mineralocorticoid Receptor Antagonist, IV: Intravenous, KCCQ: Kansas City Cardiomyopathy Questionnaire, KCCQ-CSS: Kansas City Cardiomyopathy Questionnaire Clinical Summary Score, HRQoL: Health-Related Quality of Life, BP: Blood Pressure, SBP: Systolic Blood Pressure, HFpEF: Heart Failure with preserved Ejection Fraction, HFrEF: Heart Failure with reduced Ejection Fraction, EMPERIAL: Empagliflozin in Heart Failure With Reduced or Preserved Ejection Fraction: Functional Capacity and Patient-Reported Outcomes in a Randomized Controlled Trial

Author	Study year	Study design	Sample size	Definition of preserved EF	Follow-up	Key findings	Conclusions
Abraham et al. [8]	2021	RCT	EMPERIAL-Reduced: 312 patients; EMPERIAL-Preserved: 315 patients	LVEF > 40%	12 weeks	Primary endpoint (6-minute walk test distance) not met in both trials. No significant improvement in secondary endpoints. Exploratory analyses suggested benefits in congestion score and diuretic use in EMPERIAL-Reduced only.	Empagliflozin was safe but did not improve exercise capacity in HFpEF. Potential benefits for symptom relief and congestion in HFrEF but not HFpEF.
Anker et al. [6]	2021	RCT	5988 patients (2997 empagliflozin, 2991 placebo)	LVEF > 40%	Median 26.2 months	Reduced composite risk of CV death or HF hospitalization (HR 0.79, 95% CI 0.69-0.90, P<0.001). Lower HF hospitalizations (HR 0.71, 95% CI 0.60-0.83). Slowed eGFR decline (-1.25 vs. -2.62 ml/min/1.73 m² per year, P<0.001). No significant reduction in CV mortality.	Empagliflozin reduced HF hospitalizations and slowed renal decline in HFpEF patients, regardless of diabetes status, without significant effect on CV mortality.
Packer et al. [16]	2021	RCT	5988 patients (2997 empagliflozin, 2991 placebo)	LVEF > 40%	Median 26.2 months	Significantly reduced combined risk of CV death, HF hospitalization, or urgent care visits requiring IV treatment (HR: 0.77; 95% CI: 0.67-0.87; P<0.0001). Reduced total HF hospitalizations requiring intensive care. Fewer patients required outpatient intensification of diuretics. Effect consistent across subgroups but attenuated with EF ≥60%.	Empagliflozin led to early and sustained reduction in worsening HF events in HFpEF patients, supporting its role in treatment.
Anker et al. [9]	2022	Post-hoc analysis	5988 patients (2997 empagliflozin, 2991 placebo)	LVEF > 40%	Median 26.2 months	Primary endpoint (CV death or worsening HF) reduced by 24% overall and 28% in EF <60%. Renal composite endpoint showed 40% risk reduction in EF <60% (p=0.037).	Using alternative endpoints, empagliflozin reduced CV death/worsening HF in HFpEF. Renal benefits became significant when excluding patients with LVEF ≥60%.
Butler et al. [12]	2022	RCT	5988 patients (2997 empagliflozin, 2991 placebo)	LVEF > 40%	Median 26.2 months	Reduced CV death or HF hospitalization similarly in both sexes. No significant sex-based differences in primary or secondary outcomes. Comparable improvement in KCCQ in men and women.	Empagliflozin is effective in both men and women with HFpEF. Treatment decisions should be made independently of sex.
Butler et al. [17]	2022	RCT	5988 patients (2997 empagliflozin, 2991 placebo)	LVEF > 40%	12, 32, and 52 weeks	Reduced risk of CV death or HF hospitalization across all baseline KCCQ scores (HR: 0.70-0.83). Improved HRQoL measures including KCCQ scores. Benefits sustained over 1 year. Higher odds of improvement and lower odds of deterioration.	Empagliflozin significantly improved HRQoL and reduced major HF outcomes in HFpEF patients. Benefits were consistent across patient populations and persisted over 1 year.
Ferreira et al. [14]	2022	RCT	5988 patients (2997 empagliflozin, 2991 placebo)	LVEF > 40%	52 weeks	Reduced HF hospitalizations, with more pronounced effect in MRA nonusers than users. No significant difference in CV death between MRA users/nonusers. Reduced hyperkalemia occurrence.	Benefits in reducing HF hospitalizations were stronger in MRA nonusers. Empagliflozin did not significantly affect CV death rates. Reduced hyperkalemia risk supports safe combination with MRAs.
Filippatos et al. [15]	2022	RCT	5988 patients (2997 empagliflozin, 2991 placebo)	LVEF > 40%	124-172 weeks	Significantly reduced risk of CV death or first HF hospitalization, irrespective of diabetes status. Slowed eGFR decline more in diabetic patients. No significant effect on all-cause mortality. No increased risk of hypoglycemia in non-diabetic patients.	Empagliflozin improves HF outcomes and slows kidney function decline regardless of diabetes status. Glycemic status should not influence the decision to use empagliflozin for HFpEF.
Böhm et al. [11]	2022	RCT	5988 patients (2997 empagliflozin, 2991 placebo)	LVEF > 40%	Median 26.2 months	Significantly reduced composite of CV death and HF hospitalization across all age groups. Improved KCCQ-CSS scores. Slowed eGFR decline. No significant differences in adverse events between groups across ages.	Empagliflozin effectively reduced HF hospitalizations and improved symptoms regardless of patient age, with good tolerability even in elderly populations.
Böhm et al. [10]	2023	RCT	5988 patients (2997 empagliflozin, 2991 placebo)	LVEF > 40%	Median 26.2 months	Empagliflozin reduced CV death and HF hospitalization regardless of baseline systolic BP. No significant interaction between BP and treatment effects. Similar adverse events across SBP groups.	Empagliflozin is effective and safe for HFpEF patients regardless of baseline systolic BP. Low systolic BP should not be a barrier to treatment initiation.
Coats et al. [13]	2024	RCT	5988 patients (2997 empagliflozin, 2991 placebo)	LVEF > 40%	Median 26.2 months	Increasing frailty associated with worse CV outcomes. Empagliflozin reduced CV death or HF hospitalization across all frailty categories, though effect diminished in severely frail patients. Improved HRQoL and reduced frailty over time.	Empagliflozin demonstrated efficacy across all frailty levels, though benefits were attenuated in severely frail individuals. Treatment improved frailty status and was well-tolerated in older, frail HFpEF patients.

Several post-hoc and subgroup analyses of the EMPEROR-Preserved trial were included, focusing on specific patient characteristics such as sex, age, blood pressure status, frailty, and diabetes status. These analyses provided additional insights into the effectiveness of empagliflozin across various patient populations. Most studies evaluated key outcomes including cardiovascular mortality, heart failure hospitalizations, and quality of life measures. Some studies also assessed renal outcomes, with follow-up ranging from 52 weeks to 172 weeks. The evidence consistently supported empagliflozin's benefit in reducing heart failure hospitalizations across diverse HFpEF populations, though effects on cardiovascular mortality were less pronounced.

Discussion

This systematic review evaluated the clinical outcomes associated with empagliflozin in patients with HFpEF through analysis of 11 studies, predominantly derived from the landmark EMPEROR-Preserved trial. The findings consistently demonstrate that empagliflozin significantly reduces heart failure hospitalizations across diverse patient populations with HFpEF. However, a notable observation is the lack of significant effect on cardiovascular mortality, highlighting a key difference compared to its established benefits in HFrEF.

The primary efficacy of empagliflozin appears to be in reducing HF hospitalizations, with a hazard ratio of 0.71 (95% CI 0.60-0.83) as reported by Anker et al. in the main EMPEROR-Preserved trial [6]. This benefit was consistent across multiple subgroup analyses, including those based on sex, age, diabetes status, blood pressure, and frailty levels [10-14,17]. The consistency of this finding strengthens the evidence for empagliflozin as a valuable addition to the HFpEF treatment armamentarium, especially considering the historically limited therapeutic options for this condition. The reduction in hospitalizations not only represents an important clinical benefit for patients but also has significant implications for healthcare resource utilization and costs.

Regarding cardiovascular mortality, Anker et al. did not demonstrate a statistically significant reduction (HR 0.91, 95% CI 0.76-1.09) [6]. This contrasts with findings from studies of empagliflozin in HFrEF, where both morbidity and mortality benefits have been observed. The differential impact on mortality between HFpEF and HFrEF populations may reflect the greater heterogeneity in HFpEF pathophysiology, suggesting that certain subgroups might derive greater mortality benefit than others. Future research should aim to identify specific HFpEF phenotypes that may respond more favorably to empagliflozin in terms of mortality reduction. The evaluation of renal outcomes revealed that empagliflozin slowed the decline in estimated glomerular filtration rate (eGFR) compared to placebo, with a difference of -1.25 vs. -2.62 ml/min/1.73 m² per year (P<0.001) [6,9]. This renoprotective effect is consistent with findings from studies of SGLT2 inhibitors in diabetic kidney disease and suggests that empagliflozin may offer additional benefits beyond heart failure symptom improvement. The renal protection appeared more pronounced in patients with diabetes, though it was significant in non-diabetic patients as well, indicating a benefit independent of glycemic control [6,9-11,15].

Health-related quality of life (HRQoL) improvements, as measured by the Kansas City Cardiomyopathy Questionnaire (KCCQ), were consistently observed across studies that assessed this outcome. Butler et al. demonstrated that empagliflozin improved both KCCQ Total Symptom Score and Overall Summary Score, with the benefits sustained over one year [17]. These improvements in patient-reported outcomes are particularly important in HFpEF, where symptom burden and functional limitations significantly impact daily life. However, it is worth noting that the EMPERIAL-Preserved trial did not show significant improvements in exercise capacity as measured by the 6-minute walk test, suggesting that subjective symptom improvement may not always translate to objective functional capacity enhancement [8].

The subgroup analyses provide valuable insights into the efficacy of empagliflozin across different patient populations. The consistent benefit observed regardless of sex (Butler et al.), age (Böhm et al.), and diabetes status (Filippatos et al.) supports a broad clinical application of empagliflozin in HFpEF management [11,12,15]. The attenuated benefit in patients with LVEF ≥60%, as noted by Packer et al., and in severely frail individuals, as reported by Coats et al., suggests that patient selection may still play a role in optimizing treatment outcomes [13,16].

The safety profile of empagliflozin was favorable across all studies, with no new safety concerns identified. The drug was well-tolerated even in elderly and frail populations, which is particularly relevant given the demographic characteristics of HFpEF patients. The EMPERIAL-Preserved trial and subsequent analyses found no significant increase in adverse events such as hypotension, volume depletion, or acute renal failure, even in patients with lower baseline systolic blood pressure or advanced age [8].

An interesting finding from Ferreira et al. was the interaction between empagliflozin and mineralocorticoid receptor antagonist (MRA) use [14]. The benefit of empagliflozin in reducing heart failure hospitalizations was more pronounced in MRA non-users than users, and empagliflozin reduced the occurrence of hyperkalemia, potentially enabling safer MRA use. This suggests potential considerations for sequencing therapies in HFpEF management and highlights the importance of evaluating combination strategies.

Limitations and future directions

Despite promising findings, this systematic review has several notable limitations. The predominance of data from the EMPEROR-Preserved trial and its derivatives limits the diversity of evidence sources, potentially introducing bias. Although robust, this reliance on a single large trial warrants cautious interpretation of the findings. Additionally, the definition of HFpEF used in the included studies (LVEF >40%) differs from the traditional threshold (LVEF ≥50%), potentially incorporating patients with mid-range ejection fraction who may respond differently to treatment than those with strictly preserved ejection fraction. The median follow-up period of approximately 26 months may be insufficient to fully evaluate long-term efficacy and safety, particularly regarding cardiovascular mortality and progression of renal dysfunction. Furthermore, the heterogeneity in reported outcomes and methodological approaches across studies precluded meta-analysis, limiting our ability to provide pooled effect estimates.

Future research should focus on several key areas. First, identifying specific HFpEF phenotypes that derive maximal benefit from empagliflozin through advanced phenotyping approaches may enable more personalized treatment strategies. Second, studies with longer follow-up periods are needed to assess sustained benefits and potential long-term effects. Third, investigation into optimal combination strategies with other heart failure therapies, particularly mineralocorticoid receptor antagonists and novel agents like GLP-1 receptor agonists, may elucidate synergistic effects. Additionally, mechanistic studies exploring the exact pathways by which empagliflozin improves outcomes in HFpEF would enhance our understanding of its therapeutic effects. Finally, real-world effectiveness studies across diverse patient populations and healthcare settings would complement the current evidence base, ensuring broader applicability of findings to clinical practice.

## Conclusions

This systematic review provides comprehensive evidence supporting empagliflozin as an effective therapy for HFpEF, predominantly through reduction in heart failure hospitalizations and improvement in quality of life measures. The consistency of these benefits across diverse patient subgroups, including those stratified by sex, age, diabetes status, and frailty, suggests broad applicability in clinical practice. However, the lack of significant cardiovascular mortality reduction highlights an important distinction from its effects in HFrEF and underscores the heterogeneous nature of HFpEF pathophysiology. The renoprotective effects and favorable safety profile further enhance its clinical value, particularly in older populations with multiple comorbidities who typically constitute the HFpEF demographic. Future research should focus on identifying specific HFpEF phenotypes that derive maximal benefit from empagliflozin, evaluating long-term efficacy and safety, exploring optimal combination strategies with other heart failure therapies, elucidating the precise mechanisms of action, and assessing real-world effectiveness across diverse populations.
